# Microarray-based analysis of microRNA expression in breast cancer stem cells

**DOI:** 10.1186/1756-9966-29-174

**Published:** 2010-12-31

**Authors:** Jian-guo Sun, Rong-xia Liao, Jun Qiu, Jun-yu Jin, Xin-xin Wang, Yu-zhong Duan, Fang-lin Chen, Ping Hao, Qi-chao Xie, Zhi-xin Wang, De-zhi Li, Zheng-tang Chen, Shao-xiang Zhang

**Affiliations:** 1 Cancer Institute of People's Liberation Army, Xinqiao Hospital, Third Military Medical University, Chongqing, 400037, China; 2 Department of Biochemistry and Molecular Biology, Third Military Medical University, Chongqing, 400038, China; 3 Department of Anatomy, College of Medicine, Third Military Medical University, Chongqing, 400038, PR China

## Abstract

**Background:**

This study aimed to determine the miRNA profile in breast cancer stem cells (BCSCs) and to explore the functions of characteristic BCSC miRNAs.

**Methods:**

We isolated ESA^+^CD44^+^CD24^-/low ^BCSCs from MCF-7 cells using fluorescence-activated cell sorting (FACS). A human breast cancer xenograft assay was performed to validate the stem cell properties of the isolated cells, and microarray analysis was performed to screen for BCSC-related miRNAs. These BCSC-related miRNAs were selected for bioinformatic analysis and target prediction using online software programs.

**Results:**

The ESA^+^CD44^+^CD24^-/low ^cells had up to 100- to 1000-fold greater tumor-initiating capability than the MCF-7 cells. Tumors initiated from the ESA^+^CD44^+^CD24^-/low ^cells were included of luminal epithelial and myoepithelial cells, indicating stem cell properties. We also obtained miRNA profiles of ESA^+^CD44^+^CD24^-/low ^BCSCs. Most of the possible targets of potential tumorigenesis-related miRNAs were oncogenes, anti-oncogenes or regulatory genes.

**Conclusions:**

We identified a subset of miRNAs that were differentially expressed in BCSCs, providing a starting point to explore the functions of these miRNAs. Evaluating characteristic BCSC miRNAs represents a new method for studying breast cancer-initiating cells and developing therapeutic strategies aimed at eradicating the tumorigenic subpopulation of cells in breast cancer.

## Background

Breast cancer is one of the most common cancers in women and poses a threat to women's health. Al-Hajj's research in 2003 has shown that breast cancer stem cells (ESA^+^CD44^+^CD24^-/low^, BCSCs) possessing the stem cell properties of self-renewal and multi-directional differentiation are the most fundamental contributors to drug resistance, recurrence and metastasis of breast cancer [1]. Previous studies in both breast cancer cells and tissues have shown that breast cancer stem cells are cells with an ESA^+^CD44^+^CD24^-/low ^phenotype [2,3]. We based this study on the previous findings on breast cancer stem cell phenotype and finally proved it. Research focusing on BCSCs is likely to bring revolutionary changes to our understanding of breast cancer; however, a multitude of unresolved issues remain with regard to the molecular basis of carcinogenesis. For example, what is the full nature of the involvement of BCSCs in the molecular mechanisms of tumorigenesis? Are microRNAs (miRNAs) involved in the function of BCSCs? If so, how are they involved?

As an important class of regulatory noncoding RNAs, miRNAs have been shown to play important roles in the committed differentiation and self-renewal of embryonic stem cells and adult stem cells [4]. The current release (10.0) of miRBase contains 5071 miRNA loci from 58 species [5]. miRNAs can act as oncogenes or anti-oncogenes and are involved in tumorigenesis, including chronic lymphocytic leukaemia, paediatric Burkitt's lymphoma, gastric cancer, lung cancer and large-cell lymphoma [6-8]. In Homo sapiens, miRNAs (1048 sequences in miRBase 16, Sep 10^th^, 2010) regulate more than one-third of all genes, bringing hope to studies of cancer stem cells http://www.mirbase.org/. Thus, the identification of cancer stem cell-related miRNAs would provide valuable information for a better understanding of cancer stem cell properties and even the molecular mechanisms of carcinogenesis. Here, we investigated the miRNA expression profiles of ESA^+^CD44^+^CD24^-/low ^BCSCs from the MCF-7 cell line.

## Methods

### Fluorescence-activated cell sorting (FACS) of BCSCs

The human breast cancer cell line MCF-7 was cultured in minimal essential medium (MEM) (Invitrogen, America). Cells in log phase were digested with 0.25% trypsin (Gibco, America) and washed with PBS, then stained with FITC-conjugated anti-ESA, APC-conjugated anti-CD44 and PE-conjugated anti-CD24 (BD PharMingen, America). After 30 min incubation, the cells were washed three times, and FACS (MoFlo, America) was performed to isolate the ESA^+^CD44^+^CD24^-/low ^cells.

### Colony-forming assay of BCSCs

The isolated ESA^+^CD44^+^CD24^-/low ^lineage^- ^cells were suspended in MEM supplemented with 1% FBS and washed twice with the same medium. The medium was then replaced with EpiCult™-B medium (Stemcell technologies, Canada) supplemented with 5% FBS. Subsequently, 1 × 10^4 ^BCSCs were seeded onto 2 × 10^4 ^irradiated NIH/3T3 feeder cells in 24-well plates. The mouse embryonic fibroblast cell line NIH/3T3 was cultured in DMEM (Invitrogen). As feeder layer cells, NIH/3T3 cells in log phase were exposed to ^60^Co at 50 Gy. The medium was replaced again with serum-free EpiCult™-B medium at 24 hr after seeding, and the cells were incubated in 5% CO_2 _at 37°C. The cells were supplied with fresh medium every 3 days, and colonies were observed under a microscope after 7-10 days.

### Human breast cancer xenograft assay

Eight-week-old female NOD/SCID mice were given 2.5 Gy of ^60^Co radiation, and tumor cell injections were performed 1 day after irradiation. The tumor cells were suspended in 0.2 ml of IMDM containing 10% FBS and injected into the mammary fat pad at the left armpit. The mice in the test group were injected with 0.5 × 10^3^, 1 × 10^3^, 5 × 10^3^, 1 × 10^4 ^or 5 × 10^4 ^ESA^+^CD44^+^CD24^-/low ^cells isolated by FACS, whereas the mice in the control group were injected with 1 × 10^4^, 5 × 10^4^, 1 × 10^5^, 5 × 10^5 ^or 1 × 10^6 ^MCF-7 cells. Three mice in each group were inoculated with the same amount of cells. The mice were observed for tumor growth every 10 days over 8 weeks and then sacrificed by cervical dislocation. Single cell suspensions were obtained according to our previously published protocol [9]. Subsequently, ESA^+^CD44^+^CD24^-/low ^cells were isolated from the xenograft tumor cells by FACS and injected into the mammary fat pad as described above. All animal procedures were carried out with the approval of the Animal Ethics Committee of the Third Military Medical University.

### Immunostaining of tissue sections

Tumor tissue slides were prepared for immunohistochemistry. Epithelial membrane antigen (EMA) and smooth muscle actin (SMA), markers of luminal epithelial and myoepithelial cells, respectively, were used for immunostaining according to our previously published protocol [9]. Rabbit polyclonal anti-EMA or anti-SMA antibodies (dilution 1:500; Santa Cruz, CA) were used.

### Microarray Fabrication and miRNA hybridisation

Both miRNA microarray fabrication and hybridisation were performed as described previously [9]. Our miRNA microarray included 517 mature miRNA sequences and 122 published predicted miRNA (Pred_miR) sequences [10]. For each sample, two hybridisations were carried out, and each miRNA probe had three replicate spots on the microarray. Significance Analysis of Microarrays (SAM, version 2.1) was performed using a two class-unpaired comparison in the SAM procedure.

### Real-time RT-PCR

All primers were designed using Primer Express version 2.0 (Applied Biosystems, Foster City, CA). We followed the protocol of Chen et al. for primer design and real-time RT-PCR [11]. The primers were 5'-ctcgcttcggcagcaca-3' and 5'-aacgcttcacgaatttgcgt-3' for the U6 small nuclear RNA, which was used as an internal control. The analysed miRNAs included miR-122a, miR-188, miR-200a, miR-21, miR-224, miR-296, miR-301, miR-31, miR-373* and miR-200C.

### Bioinformatic analysis and target prediction

Three online software programs, miRanda http://microrna.sanger.ac.uk, picTar http://www.ncrna.org/KnowledgeBase/link-database/mirna_target_database, and targetscan http://www.targetscan.org, were used for bioinformatic analysis and target prediction for the miRNAs.

## Results

### Isolation and culture of ESA^+^CD44^+^CD24^-/low ^cells

**Figure 1:**
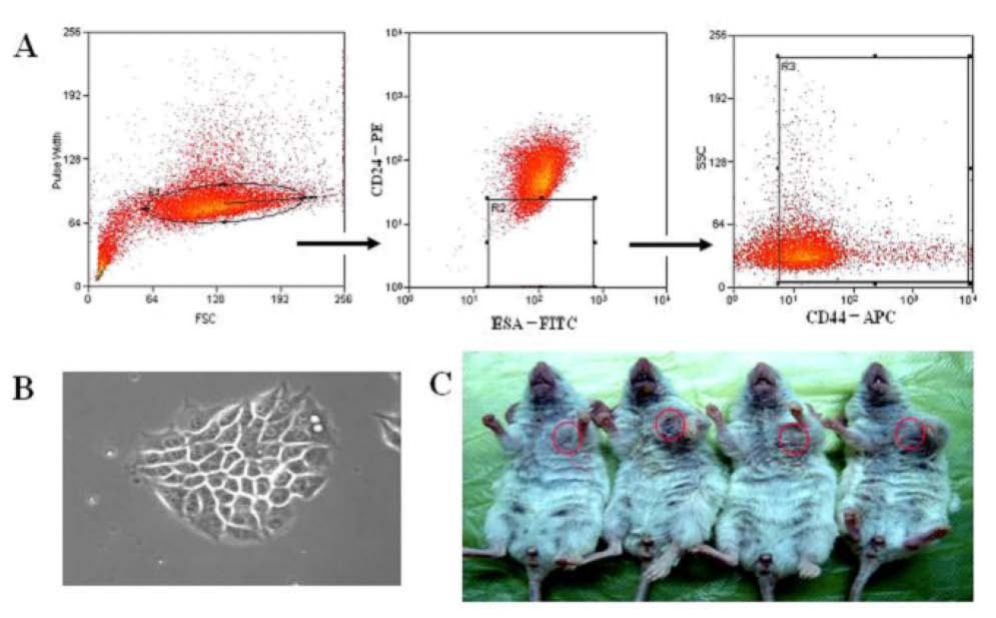
**Stem cell properties of BCSCs**. ESA^+^CD44^+^CD24^-/low^lineage^- ^human BCSCs (corresponding to 1.5% of cancer cells) were isolated by flow cytometry (A). Under an inverted phase contrast microscope, the ESA^+^CD44^+^CD24^-/low ^grew into globular colonies (B). Xenograft tumors in NOD/SCID mice are shown (C). From left to right, tumors developed from 5 × 10^5 ^and 5 × 10^6 ^MCF-7 cells and from 5 × 10^3 ^and 5 × 10^4 ^BCSCs.

The expression of ESA, CD44 and CD24 in MCF-7 cells were analyzed by flow cytometry. A 1-2% frequency of ESA^+^CD44^+^CD24^-/low^lineage^- ^cells was observed, and the cells were isolated by flow cytometry (Figure 1A). Using FACS sorting, this subpopulation of cells was highly purified (98-99% purity). To assess the clonogenic potential of these BCSCs, the cells were seeded into 24-well plates on top of irradiated NIH/3T3 feeder cells. At day 3, the number of adherent cells increased, and three to five epithelioid colonies formed. At day 6, the colonies continued to expand and spread stereoscopically. After 10 days in culture, most of the colonies contained more than 50 cells and were surrounded by floating or dead NIH/3T3 cells. Under an inverted phase contrast microscope, the ESA^+^CD44^+^CD24^-/low ^cells were observed to grow into globular colonies (Figure 1B). These cells showed no special morphological changes, however, compared with MCF-7 cells.

### Stem cell properties of ESA^+^CD44^+^CD24^-/low ^cells

We injected isolated ESA^+^CD44^+^CD24^-/low ^cells and MCF-7 cells (as a control) subcutaneously into the armpits of NOD/SCID mice. After 8 weeks, the MCF-7 cells gave rise to new tumors when ≥5 × 10^5 ^cells were injected but failed to do so at lower doses (1 × 10^5 ^cells). In contrast, the ESA^+^CD44^+^CD24^-/low ^cells formed tumors in three of three, three of three and one of three animals when 5 × 10^4^, 1 × 10^4^, and 5 × 10^3 ^cells were injected, respectively. Tumor specimens were retrieved and subsequently passaged into recipient mice. At 8 weeks after inoculation, three of three, three of three, and two of three recipient animals formed tumors when 5 × 10^4^, 1 × 10^4 ^and 5 × 10^3 ^cells were injected, respectively. Tumors were also observed in one of three animals in the control group when 5 × 10^5 ^cells were injected; however, 5 × 10^4 ^-1 × 10^5 ^cells failed to form tumors in the control group (Table 1 Figure 1C). These data indicate that ESA^+^CD44^+^CD24^-/low ^cells are tumorigenic and have up to 100- to 1000-fold greater tumor-initiating capability than MCF-7 cells.

**Table 1:** Human breast cancer xenograft assay of the ESA^+^CD44^+^CD24^-/low^ population

	Tumors-developed mice/cell-injected mice							
Injected cell number	1 × 10^6^	5 × 10^5^	1 × 10^5^	5 × 10^4^	1 × 10^4^	5 × 10^3^	1 × 10^3^	5 × 10^2^
MCF-7 cell line								
Unsorted MCF-7	3/3	1/3	0/3	0/3	0/3	-	-	-
ESA^+^CD44^+^CD24^-/low ^BCSCs	-	-	-	3/3	3/3	1/3	0/3	0/3
Xenograft tumor cells								
Unsorted breast cancer cells	3/3	1/3	0/3	0/3	-	-	-	-
ESA^+^CD44^+^CD24^-/low ^BCSCs	-	-	-	3/3	3/3	1/3	0/3	0/3

MCF-7 cells gave rise to new tumors when at least 5 × 10^5 ^cells were injected per animal but failed to do so at lower doses (10^5 ^cells). By contrast, ESA^+^CD44^+^CD24^-/low ^cells formed tumors when 5 × 10^3 ^cells were injected per animal. Tumor specimens were retrieved and subsequently passaged into recipient mice, and the same results were observed.

In addition, we tested ESA+CD44+/CD24- subpopulation variability in the murine model by FACS analysis. ESA+CD44+/CD24- subpopulation in unsorted MCF-7 xenografts remained to be 1-2%, showing little change. By contrast, ESA+CD44+/CD24- subpopulation in sorted MCF-7 xenografts were significantly enriched to 4-5%.

**Figure 2:**
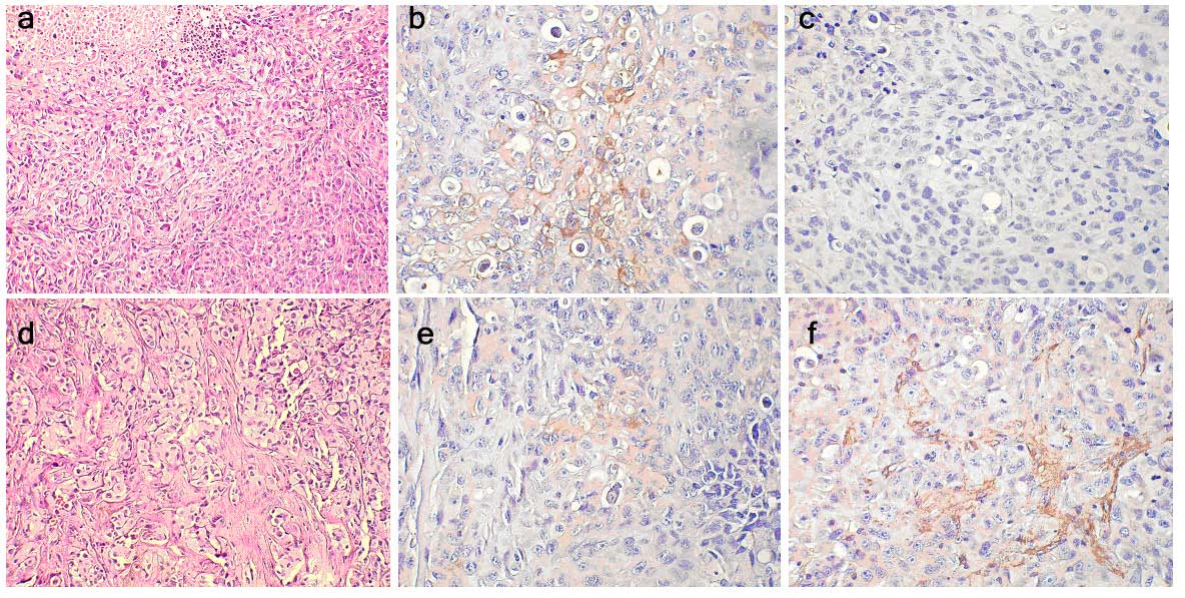
**MiRNAs expression profiles by microarray with Q-RT-PCR verification**. Haematoxylin and eosin (H&E) staining and immunohistochemical staining are shown on pathology sections of tumors implanted in NOD/SCID mice. In a, b and c, the staining showed a single cell type by H&E (100×), EMA-positive cells (200×) and SMA-negative cells (200×), respectively, for the MCF-7 group. In d, e and f, the staining showed at least two cell types by H&E (100×), EMA-positive cells (200×) and SMA-positive cells (200×), respectively, for the BCSC group.

Tumor tissue slides were prepared for H&E staining and immunohistochemical staining. The tumors in the BCSCs group were positive for both EMA and SMA, indicating that they were included of both luminal epithelial and myoepithelial cells. On the other hand, the tumors in the MCF-7 control group were positive for EMA, but negative for SMA, indicating that they were included of luminal epithelial cells, but not myoepithelial cells (Figure 2).

### MiRNA expression profiles in ESA^+^CD44^+^CD24^-/low ^BCSCs

For each cell type, the hybridisation reaction was repeated twice. The internal control U6 snRNA spots on all of the microarrays showed consistent signal strength, and the signal intensity of all of the detected spots on the replicate microarrays indicated high correlation coefficients (R = 0.9747 ± 0.0304), highlighting the reproducibility of hybridisation between the replicate microarrays(Additional file 1 Figure S1). There were 147 miRNAs in the MCF-7 cells and 102 miRNAs in the BCSCs, including predicted miRNAs (PRED_MIR), which gave a signal value above 800. The previously reported miRNA expression profile of MCF-7 cells (Ambion, USA) included 41 miRNAs (signal value ≥++). Among those miRNAs, 34 were also detected in our study, indicating a concordance rate of 82.9% (Additional file 1Table S1 S2 & S3). We compared the miRNA expression profiles of BCSCs and MCF-7 cells using a normalisation factor and clustering. A miRNA was defined as differentially expressed when a value of p < 0.05 was obtained. We identified 25 differentially expressed miRNAs that fell into two groups (fold change ≥ 4). In the first group, there were 19 miRNAs with an expression level that was four times higher in BCSCs than in MCF-7 cells: miR-122a, miR-152, miR-212, miR-224, miR-296, miR-31, miR-373*, miR-489, PRED_MIR127, PRED_MIR154, PRED_MIR157, PRED_MIR162, PRED_MIR165, PRED_MIR191, PRED_MIR207, PRED_MIR219, PRED_MIR246, PRED_MIR88 and PRED_MIR90. In the second group, there were six miRNAs with an expression level that was four times lower in BCSCs than in MCF-7 cells: miR-200a, miR-301, miR-188, miR-21, miR-181d and miR-29b.

### Validation of microarray differential expression data by real-time RT-PCR

**Figure 3:**
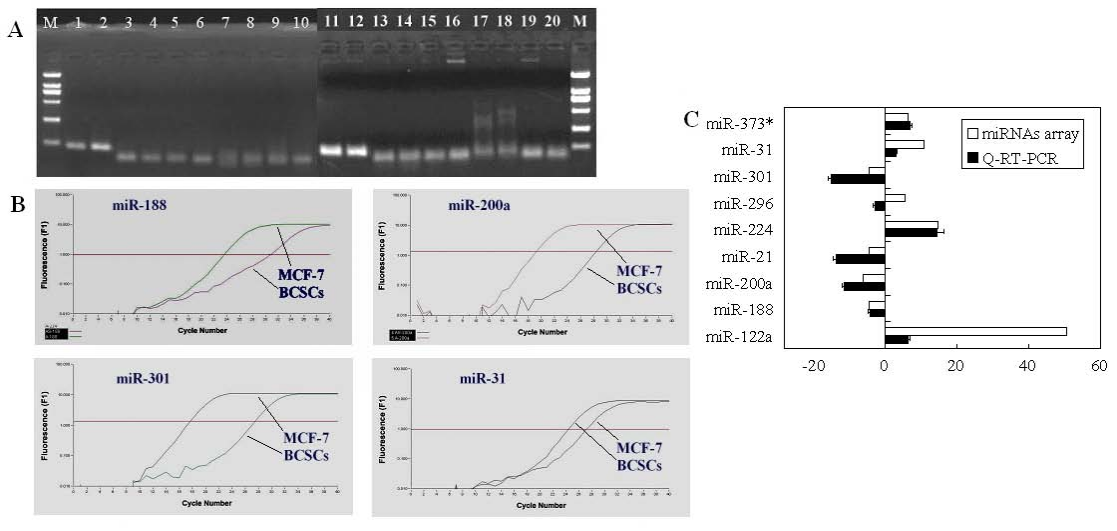
**Q-RT-PCR verification of miRNA expression**. Gel electrophoresis showed clear and specific bands for all the Q-RT-PCR reactions (A). The amplification curves in the PCR reactions were also clear. Parts of the amplification curves for miR-188, miR-200a miR-301 and miR-31 are shown (B). Ten miRNAs were compared between BCSCs and MCF-7 cells by Q-RT-PCR. Eight of the nine miRNAs tested by real-time RT-PCR gave results consistent with the microarray data, except miR-296, indicating a concordance rate of 88.89% (C).

We performed real-time RT-PCR for 10 miRNAs: miR-122a, miR-188, miR-200a, miR-21, miR-224, miR-296, miR-301, miR-31, miR-373* and miR-200C. As a negative control, miR-200C did not show obvious difference in our study. The experiments were repeated three times each. Eight of the ten miRNAs tested gave real-time RT-PCR results that were concordant with the microarray data, with miR-296 being the only exception, indicating a concordance rate of 88.89%. The electrophoretogram showed clear and specific bands for all of the real-time RT-PCR reactions, and all the amplification curves in the PCR reactions were distinct (Figure 3A). Part of amplification curves for miR-188, miR-200a miR-301 and miR-31 are shown in Figure 3B. The Q-RT-PCR results for the 10 miRNAs tested were 6.344 ± 0.402, 0.226 ± 0.513, 0.086 ± 0.514, 0.071 ± 0.503, 14.175 ± 2.033, 0.334 ± 0.587, 0.066 ± 1.008, 2.816 ± 0.328, 6.684 ± 0.548 and 0.345 ± 0.531 (expressed as the relative ratio between the Q-RT-PCR results for BCSCs and MCF-7 cells ± standard deviation). Despite little difference in the microarray results, the expression of miR-200c was found to be no more than three times lower in BCSCs than in MCF-7 (Figure 3CTable 2). Thus, the miRNA expression profiles of the BCSCs were confirmed by Q-RT-PCR.

**Table 2:** Verification The microarray data were verified by Q-RT-PCR

Name	E	CT(BCSCs)	CT(MCF-7)	ΔCT (BCSCs-MCF7)	RQ (BCSCs/U6)	RQ (MCF-7/U6)	RQ (BCSCs/MCF7)	Chip (BCSCs/MCF7)
**U6 RNA**	1.893 ± 0.087	18.307 ± 0.163	15.003 ± 0.227	3.303 ± 0.297			8.154 ± 0.516	
**miR-122a**	1.885 ± 0.098	23.650 ± 2.810	23.253 ± 2.812	0.397 ± 0.031	0.041 ± 0.007	0.006 ± 0.001	6.344 ± 0.402	50.414
**miR-188**	1.766 ± 0.036	31.103 ± 0.539	24.795 ± 0.508	6.308 ± 0.129	0.004 ± 0.003	0.015 ± 0.001	0.226 ± 0.513	0.207
**miR-200a**	1.900 ± 0.074	28.387 ± 0.261	21.253 ± 0.632	7.134 ± 0.652	0.002 ± 0.001	0.021 ± 0.017	0.086 ± 0.514	0.159
**miR-21**	1.899 ± 0.011	24.657 ± 1.325	17.263 ± 1.435	7.393 ± 0.195	0.016 ± 0.003	0.226 ± 0.051	0.071 ± 0.503	0.211
**miR-224**	1.683 ± 0.065	32.437 ± 0.400	33.497 ± 0.624	-1.060 ± 0.288	0.011 ± 0.001	0.001 ± 0.000	14.175 ± 2.033	14.491
**miR-296**	1.905 ± 0.025	27.237 ± 0.291	22.247 ± 0.468	4.990 ± 0.255	0.003 ± 0.001	0.009 ± 0.003	0.334 ± 0.587	5.242
**miR-301**	1.873 ± 0.017	27.487 ± 0.476	19.791 ± 0.619	7.696 ± 0.179	0.005 ± 0.004	0.081 ± 0.006	0.066 ± 1.008	0.205
**miR-31**	1.817 ± 0.027	27.397 ± 0.448	25.613 ± 0.634	1.783 ± 0.210	0.013 ± 0.001	0.005 ± 0.000	2.816 ± 0.328	10.700
miR-373*	1.902 ± 0.040	24.370 ± 1.438	24.060 ± 1.404	0.310 ± 0.096	0.019 ± 0.001	0.003 ± 0.000	6.684 ± 0.548	6.183
**miR-200C**	1.888 ± 0.053	24.513 ± 0.658	19.527 ± 0.938	4.987 ± 0.290	0.032 ± 0.042	0.100 ± 0.013	0.345 ± 0.531	1.720

### Bioinformatic analysis and preliminary functional analysis of BCSC-related miRNAs

Chromosome localisation, sequence analysis and target prediction of the miRNAs were carried out using online software programs. Potential tumorigenesis-related miRNAs and their possible targets were analysed. Most of these targets were oncogenes, anti-oncogenes or regulatory genes involved in miRNA processing, transcriptional regulation, signal transduction, apoptosis regulation and stem cell function and maintenance, etc. For example, there were 161 potential targets of miR-122a, including RAD21, G3BP2, CDC42BPB, SP2, GPR172B, GPR172A, MAP3K3, DR1, KHDRBS1, MAP3K12, CCNG1 and DICER1. These potential targets included oncogenes, transcription factors and genes related to DNA repair, cell cycle regulation, miRNA processing and signal transduction. The gene encoding miR-21 was located on chromosome 17, and there were 175 potential targets of miR-21, including PLAG1, PDCD4, SKI, BCL2, STAT3, PITX2, HBP1, ELF2, E2F3, SPRY1, CDC25A, N-PAC, EIF1AX, EIF2C2, RAB11A, RAB6A, RAB6C, RASGRP1, RHOB, RASA1, TPM1, TGFBI and TNFSF6, which exist exclusively in humans, mice, dogs, chimps and chickens. These potential targets included pleiomorphic adenoma genes, transcription factors, oncogenes, anti-oncogenes, and genes related to miRNA processing and signal transduction (Additional file 1 table S4).

## Discussion

There is increasing evidence for the involvement of miRNAs in mammalian biology and breast cancer. For instance, the levels of MiR-206 have been found to be higher in ERalpha-negative MB-MDA-231 cells than in ERalpha-positive MCF-7 cells [12], and enforced expression of miR-125a or miR-125b leads to coordinate suppression of ERBB2 and ERBB3 in the human breast cancer cell line SKBR3 [13]. Furthermore, MiR-27b, which is expressed in MCF-7 cells, may be one of the causes of high expression of the drug-metabolising enzyme CYP1B1 in cancerous tissues [14]. Finally, as a tumor suppressor in breast cancer cells, miR-17-5p regulates breast cancer cell proliferation by inhibiting the translation of AIB1 mRNA [15].

Research on the roles of BCSC-related miRNAs in breast cancer has great significance. Ponti [16] isolated tumorigenic breast cancer cells with stem/progenitor cell properties from a breast cancer cell line, and Huang [17] screened side population (SP) cells from a breast cancer cell line. Here, we investigated the miRNA expression profile of the ESA^+^CD44^+^CD24^-/Low ^subpopulation from the MCF-7 cell line. Real-time RT-PCR was repeated three times, and the results were concordant with microarray data for the miRNA expression profiles of BCSCs.

Recently, a few studies have reported miRNA expression in BCSCs. Shimono [18] found that 37 miRNAs were upregulated or downregulated in BCSCs compared to nontumorigenic breast cancer cells. Three clusters, miR-200c-141, miR-200b-200a-429, and miR-183-96-182, were downregulated in human BCSCs. MiR-200c was shown to be overexpressed in MCF-7 cells, leading to reduced expression of transcription factor 8 and increased expression of E-cadherin [19]. Furthermore, the downregulation of Let-7 miRNAs rather than miR-200C was previously reported for human BCSCs [20]. Let-7 regulates multiple breast cancer stem cell properties by silencing more than one target, and Let-7 miRNAs are markedly reduced in BCSCs and increase with differentiation.

We obtained miRNA expression profiles of BCSCs, providing a substantial basis for exploring the role of miRNAs in maintaining stem cell properties and the biological functions of BCSCs. Compared with previous reports, we found that miR-200C expression was about 3-fold lower in BCSCs than in MCF-7 cells as determined by Q-RT-PCR. Little change was observed in the expression of Let-7 family members, however, between BCSCs and MCF-7 cells, with the exception of Let-7e (data not shown). The discrepancies in Let-7 and miR-200C expression between studies might be related to differences in tumor histology or the genetic backgrounds of the cell lines analysed. We also detected the expression of some predicted miRNAs in the BCSCs. Given that the existence of predicted miRNAs has yet to be validated, no accurate miRNA sequence could be used to synthesise accurate primers, making real-time RT-PCR verification unavailable. Further study of the functions of these characteristic BCSC miRNAs will facilitate research into the roles of miRNAs in breast cancer.

Bioinformatic analysis and prediction programs have been the primary methods used to explore the function of miRNAs [21,22]. The genes possibly regulated by these characteristic BCSC miRNAs are involved in both tumorigenesis and stem cell maintenance. For example, miR-122a has been reported to be specific to liver tissue [23,24]; however, our results showed upregulation of miR-122a in BCSCs. The microarray data were verified by Q-RT-PCR. Furthermore, miR-122a was also detected in MCF-7 cells in the Ambion dataset. Bioinformatic analysis showed that the potential targets of miR-122a include several cancer-related genes. In previous reports, it has been shown that miR-122a plays a role in the genesis of hepatocellular carcinoma by blocking cyclin G1 expression [25]. Another study found that G3BP2, one of the potential targets of miR-122a, was more highly expressed in breast cancer tissue than in paraneoplastic tissue [26-28]. These studies indicate that miR-122a is likely to be an important gene regulatory factor in cancer cells, even cancer stem cells. Another example is miR-21, which has been reported to have extensive roles and is expressed in embryonic stem cells [29], neuronal cells [30] and several tumor tissues [31,32]. Previous studies have demonstrated that as an oncogene, miR-21 targets the tumor suppressor gene Tropomyosin 1 (TPM1)* and may indirectly regulate genes such as the proto-oncogene bcl-2, thus modulating tumorigenesis [33,34]. In this study, miR-21 expression was lower in BCSCs than in MCF-7 cells. Interestingly, target analysis of miR-21 revealed two classes of genes with opposite functions, e.g., PLAG1 (pleiomorphic adenoma gene 1) and PDCD4 (Programmed cell death 4). As a cancer-promoting gene, PLAG1 plays an essential role in the processes of adenocarcinoma formation and malignant transformation in various types of tumors [35], whereas PDCD4 is a tumor suppressor gene that inhibits neoplastic transformation and tumor cell invasion and facilitates apoptosis [36]. Several recent studies have shown that the tumor suppressor PDCD4 is a target of miR-21 [37-39]. Nevertheless, the question remains whether PLAG1 is likely to be a target of miR-21. Moreover, the potential target genes of miR-21 include several oncogenes such as RAB11A, RAB6A, RAB6C, RASGRP1, RHOB and RASA1, etc. Are these genes the true targets of miR-21? What are the mechanisms of their involvement in the genesis of breast cancer? These intriguing questions remain to be answered.

Furthermore, the prediction of potential targets for other BCSC-related miRNAs indicated overlap between the targets of different miRNAs. For example, PLAG1 was a potential target for both miR-224 and miR-200a, and the expression of miR-200a was lower in BCSCs than in MCF-7 cells. In contrast, the expression of miR-224 was higher in BCSCs than in MCF-7 cells. It is likely that the miRNAs that are over-expressed or under-expressed in BCSCs may regulate common target genes and form a miRNA gene network by cooperating or competing with each other to regulate the development of BCSCs.

Moreover, miR-301, miR-296, miR-21 and miR-373* have been reported to be expressed in human embryonic stem cells and other stem cells, indicating that these miRNAs may play a constitutive role in maintaining the biological characteristics of stem cells [40,41]. Future work should include verification of the potential targets of all of the BCSC-related miRNAs identified here.

## Conclusions

Here, we investigated the miRNA expression profile of the ESA^+^CD44^+^CD24^-/Low ^BCSC subpopulation from the MCF-7 cell line. Our identification of BCSC-related miRNAs should be a starting point to explore the functions of these miRNAs, adding a new dimension to our understanding of the complex picture of BCSCs and assisting cancer biologists and clinical oncologists in designing and testing novel therapeutic strategies.

## Competing interests

The authors declare that they have no competing interests.

## Authors' contributions

JS conceived of the study, and participated in its design and drafted the manuscript. RL participated in the study design and carried out the FACS and microarray analysis. JQ and JJ participated in the Colony-forming assay and performed human breast cancer xenograft assay. XW and YD performed the Immunostaining. FC and PH participated in the microarray analysis. QX and ZW performed the Real-time RT-PCR. DL helped with the statistical analysis and manuscript drafting.ZC and SZ conceived of the study, and participated in its design and coordination and helped to draft the manuscript. All authors have read and approved the final manuscript.

## Supplementary Material

Additional file 1 **Figure S1- MiRNA microarray for MCF-7 cells & BCSCs**. The figure shows one array of the two hybridisations for MCF-7 cells & BCSCs. a and b show microarrays for MCF-7 cells, and c and d show microarrays for BCSC cells. **Table S1-MiRNAs microarray- based miRNAs expression profile of MCF-7 cells (signal value ≥800)**. The table shows the miRNAs expression profile of MCF-7 cells obtained through miRNAs microarray. **Table S2- MiRNAs microarray- based miRNAs expression profile of ESA+CD44+CD24-/low cells (signal value ≥800)**. The table shows the miRNAs expression profile of ESA+CD44+CD24-/low cells obtained through miRNAs microarray. **Table S3- MiRNA target prediction**. The table shows predicted targets for miR-21 and miR-122a, and the primary functions of the target genes. **Table S4- MiRNAs expression profile of MCF-7 cell from Ambion (signal value ≥++)**. The table shows MiRNAs expression profile of MCF-7 cells detected by Ambion. Click here for file
