# Supplementary material for: Microarray-based analysis of microRNA expression in breast cancer stem cells
Source: J Exp Clin Cancer Res. 2010 Dec 31;29(1):174. doi: 10.1186/1756-9966-29-174 (PMC3022679; doi:10.1186/1756-9966-29-174)
Supplement: Additional file 1 — Figure S1- MiRNA microarray for MCF-7 cells & BCSCs. The figure shows one array of the two hybridisations for MCF-7 cells & BCSCs. a and b show microarrays for MCF-7 cells, and c and d show microarrays for BCSC cells. Table S1-MiRNAs microarray- based miRNAs expression profile of MCF-7 cells (signal value ≥800). The table shows the miRNAs expression profile of MCF-7 cells obtained through miRNAs microarray. Table S2- MiRNAs microarray- based miRNAs expression profile of ESA+CD44+CD24-/low cells (signal value ≥800). The table shows the miRNAs expression profile of ESA+CD44+CD24-/low cells obtained through miRNAs microarray. Table S3- MiRNA target prediction. The table shows predicted targets for miR-21 and miR-122a, and the primary functions of the target genes. Table S4- MiRNAs expression profile of MCF-7 cell from Ambion (signal value ≥++). The table shows MiRNAs expression profile of MCF-7 cells detected by Ambion. [file 1756-9966-29-174-S1.DOC]

**
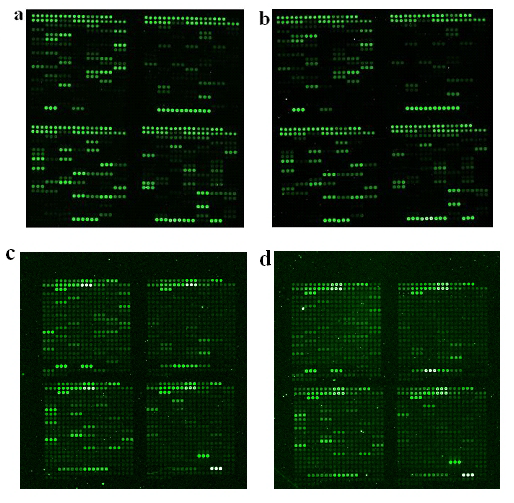
**

**Figure S1.**  For each of cell type, one array of the two hybridisations is shown. a and b show microarrays for MCF-7 cells, and c and d show microarrays for BCSCs.

**Table S1. MiRNAs microarray- based miRNAs expression profile of MCF-7 cells (signal value ≥800)**

| let-7a | miR-148a | miR-192 | miR-23b | miR-331 | miR-489 | miR-99b |
| --- | --- | --- | --- | --- | --- | --- |
| let-7b | miR-148b | miR-193a | miR-24 | miR-335 | miR-494 | PRED_MIR112 |
| let-7c | miR-151 | miR-193b | miR-25 | miR-339 | miR-497 | PRED_MIR154 |
| let-7d | miR-154* | miR-195 | miR-26a | miR-342 | miR-501 | PRED_MIR165 |
| let-7e | miR-155 | miR-198 | miR-26b | miR-345 | miR-505 | PRED_MIR166 |
| let-7f | iR-15b | miR-19a | miR-27a | miR-34a | miR-509 | PRED_MIR186 |
| let-7g | miR-16 | miR-19b | miR-27b | miR-34b | miR-516-3p | PRED_MIR189 |
| let-7i | miR-17-3p | miR-200a | miR-28 | miR-361 | miR-518f | PRED_MIR191 |
| miR-101 | miR-17-5p | miR-200b | miR-296 | miR-362 | miR-520a | PRED_MIR194 |
| miR-103 | miR-181a | miR-200c | miR-29a | miR-365 | miR-520b | PRED_MIR206 |
| miR-106a | miR-181b | miR-202 | miR-29b | miR-370 | miR-520c | PRED_MIR207 |
| miR-106b | miR-181d | miR-203 | miR-29c | miR-373 | miR-520d | PRED_MIR213 |
| miR-107 | miR-182 | miR-20a | miR-301 | miR-373* | miR-520e | PRED_MIR220 |
| miR-125a | miR-183 | miR-20b | miR-302c* | miR-374 | miR-520f | PRED_MIR234 |
| miR-126 | miR-184 | miR-21 | miR-30a-5p | miR-375 | miR-523 | PRED_MIR240 |
| miR-128a | miR-185 | miR-210 | miR-30b | miR-422a | miR-7 | PRED_MIR246 |
| miR-128b | miR-186 | miR-214 | miR-30c | miR-422b | miR-92 | PRED_MIR257 |
| miR-130a | miR-188 | miR-22 | miR-30d | miR-423 | miR-93 | PRED_MIR258 |
| miR-130b | miR-18a | miR-221 | miR-30e-5p | miR-424 | miR-96 | PRED_MIR30 |
| miR-134 | miR-18b | miR-222 | miR-320 | miR-429 | miR-98 | PRED_MIR88 |
| miR-141 | miR-191 | miR-23a | miR-324-5p | miR-432 | miR-99a | PRED_MIR90 |

**Table S2. MiRNAs microarray- based miRNAs expression profile of ESA+CD44+CD24-/low cells (signal value ≥800)**

| let-7a | miR-125b | miR-182 | miR-21 | miR-30b | miR-92 | PRED_MIR165 |
| --- | --- | --- | --- | --- | --- | --- |
| let-7b | miR-126 | miR-183 | miR-210 | miR-30c | miR-93 | PRED_MIR166 |
| let-7c | miR-128a | miR-185 | miR-212 | miR-30d | miR-99a | PRED_MIR189 |
| let-7d | miR-134 | miR-18a | miR-22 | miR-320 | miR-99b | PRED_MIR191 |
| let-7e | miR-138 | miR-191 | miR-23a | miR-331 | PRED_MIR104 | PRED_MIR206 |
| let-7f | miR-139 | miR-192 | miR-23b | miR-342 | PRED_MIR112 | PRED_MIR207 |
| let-7g | miR-141 | miR-193b | miR-24 | miR-34a | PRED_MIR114 | PRED_MIR213 |
| let-7i | miR-148a | miR-195 | miR-25 | miR-373* | PRED_MIR127 | PRED_MIR219 |
| miR-103 | miR-152 | miR-198 | miR-26a | miR-489 | PRED_MIR143 | PRED_MIR240 |
| miR-106a | miR-155 | miR-19b | miR-26b | miR-496 | PRED_MIR145 | PRED_MIR246 |
| miR-106b | miR-15a | miR-200b | miR-27a | miR-518f | PRED_MIR146 | PRED_MIR88 |
| miR-107 | miR-15b | miR-200c | miR-27b | miR-520b | PRED_MIR154 | PRED_MIR90 |
| miR-122a | miR-16 | miR-202 | miR-296 | miR-520e | PRED_MIR157 |  |
| miR-124a | miR-17-5p | miR-203 | miR-29a | miR-523 | PRED_MIR160 |  |
| miR-125a | miR-181b | miR-20a | miR-30a-5p | miR-7 | PRED_MIR162 |  |

**Table S3. MiRNA target prediction**

| **miRNAs** | **target prediction** | **Abrrev.** | **Primary Function** |
| --- | --- | --- | --- |
| **miR-21** | pleiomorphic adenoma gene 1 | PLAG1 | adenocarcinoma transformation |
|  | programmed cell death 4 (neoplastic transformation inhibitor) | PDCD4 | pro-apoptosis |
|  | v-ski sarcoma viral oncogene homolog | SKI | oncogene |
|  | B-cell CLL/lymphoma 2 | BCL2 | anti-oncogene |
|  | signal transducer and activator of transcription 3 | STAT3 | Signal transduction |
|  | paired-like homeodomain transc ription factor 2 | PITX2 | transcription factor |
|  | HMG-box transcription factor 1 | HBP1 | transcription factor |
|  | E74-like factor 2 (ets domain transcription factor) | ELF2 | transcription factor |
|  | E2F transcription factor 3 | E2F3 | transcription factor |
|  | sprouty homolog 1, antagonist of FGF signaling | SPRY1 | transcription factor |
|  | cell division cycle 25A | CDC25A | cell differentiation |
|  | cytokine-like nuclear factor n-pac | N-PAC | cytokine |
|  | eukaryotic translation initiation factor 1A, X-linked | EIF1AX | Translation regulation |
|  | eukaryotic translation initiation factor 2C, 2 | EIF2C2 | miRNA processing |
|  | RAB11A, member RAS oncogene family | RAB11A | oncogene |
|  | RAB6A, member RAS oncogene family | RAB6A | oncogene |
|  | RAB6C, member RAS oncogene family | RAB6C | oncogene |
|  | RAS guanyl releasing protein 1 | RASGRP1 | oncogene |
|  | ras homolog gene family, member B | RHOB | oncogene |
|  | RAS p21 protein activator | RASA1 | oncogene |
|  | tumor suppressor gene tropomyosin 1 | TPM1 | anti-oncogene |
|  | transforming growth factor, beta- induced | TGFBI | transcription factor |
|  | tumor necrosis factor (ligand) superfamily, member 6 | TNFSF6 | cytokine |
| **miR-122a** | RAD21 homolog | RAD21 | DNA repair |
|  | Ras-GTPase activating protein SH3 domain-binding protein 2 | G3BP2 | oncogene |
|  | CDC42 binding protein kinase beta (DMPK-like) | CDC42BPB | cell cycle |
|  | Sp2 transcription factor | SP2 | transcription factor |
|  | G protein-coupled receptor 172B | GPR172B | Gene regulation |
|  | G protein-coupled receptor 172A | GPR172A | Gene regulation |
|  | mitogen-activated protein kinase kinase kinase 3 | MAP3K3 | signal transduction |
|  | down-regulator of transcription 1, TBP-binding | DR1 | transcription factor |
|  | KH domain containing, signal transduction associated 1 | KHDRBS1 | signal transduction |
|  | mitogen-activated protein kinase kinase kinase 12 | MAP3K12 | signal transduction |
|  | cyclin G1 | CCNG1 | cell cycle |
|  | Dicer1, Dcr-1 homolog | DICER1 | miRNA processing |

**Table S4 - M**iRNAs expression profiles of MCF-7 cells from Ambion (signal value ≥++)

| let-7A | miR-130A | miR-19A | miR-25 | miR-34A | miR-18 | miR-20 |
| --- | --- | --- | --- | --- | --- | --- |
| let-7G | miR-155 | miR-200B | miR-27A | miR-7 | miR-191 | miR-21 |
| miR-1 | miR-16 | miR-22 | miR-296 | miR-93 | miR-192 | miR-106A |
| miR-103 | miR-182 | miR-221 | miR-30A | miR-99A | miR-26A | miR-194 |
| miR-107 | miR-198 | miR-23B | miR-320 | miR-140 | miR-92 | miR-321 |
| miR-122A | miR-199A-AS | miR-24 | miR-342 | miR-145 | miR-17-3P |  |

Among them, miR-1, miR-122A, miR-199A-AS, miR-140, miR-145, miR-194 and miR-321 were not detected in the present study. Compared with the Ambion data, 34 miRNAs were detected in our data set with a concordance rate of 82.9%.
